# Does exposure to startle impact voluntary reaching movements in individuals with severe-to-moderate stroke?

**DOI:** 10.1007/s00221-020-06005-4

**Published:** 2021-01-03

**Authors:** Marziye Rahimi, Zoe Swann, Claire F. Honeycutt

**Affiliations:** 1 Ira A. Fulton Schools of Engineering, Arizona State University, 699 S Mill Ave, Tempe, AZ 85281 USA; 2 School of Life Sciences, Arizona State University, 427 E Tyler Mall, Tempe, AZ 85281 USA; 3 School of Biological and Health Science Engineering, Arizona State University, 501 E Tyler Mall, Tempe, AZ 85287 USA; 4 Arizona State University, Mailcode 9709, 611 E Orange St, Tempe, AZ 85281 USA

**Keywords:** Startle, Rehabilitation, Severe stroke, Point-to-point reaching, Abnormal flexor activity

## Abstract

When movements of individuals with stroke (iwS) are elicited by startling acoustic stimulus (SAS), reaching movements are faster, further, and directed away from the body. However, these startle-evoked movements also elicit task-inappropriate flexor activity, raising concerns that chronic exposure to startle might also induce heightened flexor activity during voluntarily elicited movement. The objective of this study is to evaluate the impact of startle exposure on voluntary movements during point-to-point reaching in individuals with moderate and severe stroke. We hypothesize that startle exposure will increase task-inappropriate activity in flexor muscles, which will be associated with worse voluntarily initiated reaching performance (e.g. decreased distance, displacement, and final accuracy). Eleven individuals with moderate-to-severe stroke (UEFM = 8–41/66 and MAS = 0–4/4) performed voluntary point-to-point reaching with 1/3 of trials elicited by an SAS. We used electromyography to measure activity in brachioradialis (BR), biceps (BIC), triceps lateral head (TRI), pectoralis (PEC), anterior deltoid (AD), and posterior deltoid (PD). Conversely to our hypothesis, exposure to startle did not increase abnormal flexion but rather antagonist activity in the elbow flexors and shoulder horizontal adductors decreased, suggesting that abnormal flexor/extensor co-contraction was reduced. This reduction of flexion led to increased reaching distance (18.2% farther), movement onset (8.6% faster), and final accuracy (16.1% more accurate) by the end of the session. This study offers the first evidence that exposure to startle in iwS does not negatively impact voluntary movement; moreover, exposure may improve volitionally activated reaching movements by decreasing abnormal flexion activity.

## Introduction

When paired with a task goal, a loud, startling sound can result in enhanced movement parameters for individuals with stroke (iwS) (Honeycutt et al. 2015; Rahimi and Honeycutt 2020; Honeycutt and Perreault 2012, 2013; Coppens et al. 2018; Ossanna et al. 2019). Specifically, previous studies showed that startle-elicited reaching movements preceded by a startling acoustic stimulus (SAS) (Rahimi and Honeycutt 2020; Carlsen et al. 2004; Marinovic et al. 2016; Davis et al. 1982; Groves et al. 1974; Hammond 1973; Davis and Gendelman 1977; Nonnekes et al. 2014) are significantly faster (Rahimi and Honeycutt 2020; Davis and Gendelman 1977; Nonnekes et al. 2014; Honeycutt and Perreault 2014) and further (Rahimi and Honeycutt 2020; Ossanna et al. 2019; Carlsen and Maslovat 2019; Castellote and Valls-Sole 2015). Startle-evoked hand extension in iwS also results in faster and larger muscle activity (Honeycutt et al. 2015).

While provocative, there are several confounding factors that lead to diminished enthusiasm for this novel implementation. First, startle-evoked movements in iwS are interrupted by functionally inappropriate activation of the flexors during extension that increases with impairment and spasticity levels (Rahimi and Honeycutt 2020; Honeycutt and Perreault 2012, 2014). Specifically, when startle is used to initiate movement, heightened coactivation of antagonist flexor muscles interrupts agonist extensor muscles (e.g. triceps) leading to higher errors during point-to-point reaching tasks. The high error rate despite larger reaching movement indicates that reaching movements are not always directed towards the intended target (Rahimi and Honeycutt 2020; Castellote and Valls-Sole 2015). Some have argued this abnormal coupling is the result of increased reliance on brainstem structures (McPherson et al. 2018a, b; Ellis et al. 2012). If true, long-term exposure to startle may induce plastic changes that could lead to increased abnormal flexor activity during voluntarily initiated movement. IwS are already afflicted with abnormal flexor synergies that significantly degrade movement (Twitchell 1951; Beer et al. 1999). Thus, it is unclear if startle is a viable, or even advisable, rehabilitation tool. Furthermore, previous work has only evaluated startle-evoked movement (Honeycutt and Perreault 2012; Ossanna et al. 2019; Carlsen et al. 2004; McPherson et al. 2018a; Kirkpatrick et al. 2018; Marinovic and Tresilian 2016). No one has evaluated the impact of startle exposure on voluntarily elicited movement. Due to safety concerns, before proceeding to a randomized control trial evaluating training effects, it is prudent to perform a preliminary analysis to determine if exposure to startle has a maladaptive effect on voluntary movement leading to task-inappropriate flexor synergies.

The objective of this study is to evaluate the impact of exposure to startle on voluntary movements (non-startle-evoked) during point-to-point reaching in individuals with moderate and severe stroke. Specifically, we evaluate voluntary initiated muscle activity, reaching distance, movement displacement, movement onset, deviation from linearity, and final accuracy of reaching movements. We hypothesize that exposure to startle will increase task-inappropriate flexor activity, which will negatively impact voluntarily initiated reaching movement. We expect an increase in task-inappropriate EMG activity in the flexor muscles brachioradialis (BR), biceps (BIC), pectoralis (PEC), anterior deltoid (AD), which will be associated with worse voluntarily initiated reaching performance marked by decreased activity in extensors posterior deltoid (PD), triceps lateral head (TRI), leading to decreased distance, displacement, and final accuracy.

## Methods

### Subjects

Eleven individuals (age = 48 ± 19 years) with chronic severe to moderate stroke (upper extremity Fugl–Meyer = 8–41/66) and no-to-severe spasticity (modified ashworth = 0–4/4) participated in this study (Table 1). Inclusion/exclusion criteria included no injury to arm/shoulder in the past 6 months, at least 6 months post-stroke, no hearing loss/sensitivity, no dizzy or fainting spells, no seizure or heart attacks, measurable impairment in the upper extremity, and could not be pregnant. This study was approved by Arizona State University’s Institutional Review Board STUDY00002440. Subjects were informed of potential risks prior to participation in the study and verbal/written consent was obtained.

**Table 1:** Summary of subjects’ characteristics

Number	Sex	Age (years)	Duration of stroke (years)	Paretic arm	UEFM score	Modified Ashworth score			
						Shoulder flexion	Shoulder extension	Elbow extension	Elbow flexion
1	M	77	3.7	R	25	0	0	3	3
2	M	59	5.3	L	41	0	1	0	1+
3	M	36	10.4	R > L^a^	31	0	0	3	2
4	F	39	3.7	R	19	3	3	3	3
5	M	51	1.1	L	24	0	1	3	3
6	M	28	0.5	R	35	0	0	2	2
7	M	71	12.4	L	8	1	1	3	4
8	F	49	1.2	L	11	1	0	2	2
9	F	65	7.8	L	11	0	1	1+	0
10	F	19	0.8	L > R	14	0	0	0	1
11	F	33	13.5	R	11	1	1	1	1

*UEFM* Upper Extremity Fugl-Meyer

^a^R > L means both arms are impaired, but the right arm is more impaired, therefore, the UEFM and Modified Ashworth Scales are measured for the right arm

### Protocol

Ag/Cl surface electrodes [MVAP Medical Supplies, Newbury Park, CA, USA] were used to record activity from the brachioradialis (BR), biceps (BIC), triceps lateral head (TRI), pectoralis (PEC), anterior deltoid (AD), and posterior deltoid (PD), left (LSCM) and right (RSCM) sternocleidomastoid muscles. EMG signals were amplified by the Bortec AMT-8 system [Bortec Biomedical, Calgary, Alberta, Canada]. This system has a bandwidth of 10–1000 Hz, an input impedance of 10GΩ, and a common mode rejection ratio of 115 dB at 60 Hz. Electromyography (EMG) data were recorded at gain of 1500 and frequency of 3000 Hz by a 32-channel, 16-bit data acquisition system [NI USB-6363, National Instrumentation, Austin, TX].

For this study, an InMotion2 Interactive Therapy System (Interactive Motion Technologies, Inc, Watertown, MA 02472 USA) was used to record time and position data for the point-to-point reaching tasks performed by the subject at a sampling frequency of 1000 Hz. The InMotion2 system is a commercial version of the MIT-Manus and is designed for use in a clinical environment (Hogan, et al. 1994). Subjects’ arms rested on a custom-made arm support which was attached to the robot arm and had minimum friction with the table.

**Fig. 1:**
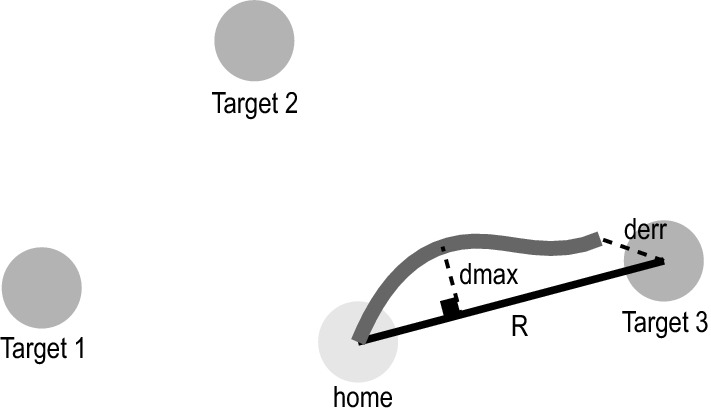
Target positions. The locations of target and home circles for the right arm are presented. The left arm targets were the mirror image of these targets. R was the distance between the home circle and the target circles (black line). The gray line is the movement trajectory from the start point to the end point. *d*_max_ is the maximum distance between the movement trajectory and the axis connecting the home to the target. *d*_err_ is the distance between the target and the end point

Subjects were asked to do a point-to-point reaching task. Subjects sat comfortably in the experimental chair with an initial arm position of shoulder abduction at 70°, shoulder flexion at 40°, and elbow angle at 90° (all ± 5° to subjects comfort). Trunk movement was minimized by using straps across the subject’s chest, so that their shoulder position was fixed and their trunk movement was minimum. They were instructed to perform point-to-point reaching movements to three target circles starting from a fixed home position. Targets were designed to cover the workspace and include mostly shoulder horizontal adduction (Target 1), mostly elbow extension (Target 3), and combination of shoulder flexion and elbow extension (Target 2) (Fig. 1). Home and target circles were displayed on a monitor, with a cursor mapping the online location of their hand as visual feedback. The distance between home and target circles was proportional to the subjects’ arm lengths. This distance was calculated using the following formula for each subject: $R = { }\frac{{\sin \left( {70^\circ } \right) \times {\text{length of upper arm}} + {\text{length of forearm}}}}{5}$. Reach targets were an average of 11.06 ± 0.25 cm.

Subjects were asked to move following two soft (80 dB) auditory sounds. The instruction was to plan to move after the first sound (GET READY) and reach as fast and accurately as possible after hearing the second sound (GO). GO cues were delivered between 2 and 3 s after the GET READY cues to prevent anticipation. Prior to the main session, subjects practiced reaching from the fixed home position to the 3 position targets (15 times to each target) with visual feedback to make sure they learned the instruction. These trials were not included in the analysis.

Following practice, the online visual feedback of the cursor was removed, in order to be consistent with previous literature (Schaefer et al. 2007, 2009a, b,  2012). Individuals with stroke tend to have deficits during the finishing phase of point-to-point reaching task especially in absence of visual feedback (Schaefer et al. 2009a). Therefore, removal of the visual feedback create more space for final error in these individuals. The cursor disappeared as soon as subjects left the home circle and reappeared half a second after they stopped to give subjects visual final point accuracy feedback. Subjects performed 135 reaches separated into blocks of 15 reaches to a single target. The order of blocks was randomized. This resulted in 45 reaches to each target. Startle was randomly applied during 1/3 of trials by replacing the soft GO cue with a startling sound of 115 dB. The startling sound was generated by Siren Speaker TS-333S, 12 V DC/1000 mA/122 dB with a duration of 0.01 s and rise time of 0.002 s. R/LSCM EMG activity prior to 120 ms during startle-evoked trials was defined as presence of startle (Schaefer et al. 2009a; Carlsen et al. 2007). Startle-evoked trials were not further evaluated in this study but have been previously reported (Rahimi and Honeycutt 2020). Modified Ashworth Scales (MAS) and Upper Extremity Fugl-Meyer Assessments (UEFM) were collected at the end of the session.

### Data analysis

The first 10 (beginning) and last 10 (end) voluntary trials to each target (i.e. the beginning and end of the session) were evaluated. EMG data were rectified and smoothed in MATLAB (R2017b) using a ten-point moving average. The following outcome measures were calculated: EMG onset, movement onset, movement distance, movement displacement, deviation from linearity, final error and EMG amplitude. EMG onset was first detected using a custom MATLAB script that detected EMG activity greater than the background activity plus 3 standard deviations. Background was calculated using 500 ms prior to the GO cue. Visual inspection and corrections were conducted by an experimenter blinded to trial type. Movement onset was defined as the time when the subject left the 1 cm HOME circle. The final point was the position that the velocity dropped down to 0.0001 m/s threshold. Movement distance was the distance traveled from the home position until the final point. The movement displacement is the absolute value of how far the final point is from the home circle. *d*_max_ was the maximum distance between the movement trajectory and the axis connecting the home to the target (Fig. 1). Deviation from linearity was defined using the following formula  $\frac{{d}_{\mathrm{max}}}{R}$ (Schaefer et al. 2009a). Final error was the distance between the final point and the center of the intended target. EMG amplitude was calculated as the maximum EMG activity over the first 70 ms preceding the onset of muscle activity. Finally, SCM + % was defined as the percentage of the startle-evoked trials with right or left SCM activity prior to 120 ms and calculated using the following formula: $100 \times \frac{{{\text{number of SCM}} + {\text{ trials}}}}{{\text{number of SAS trials}}}$. Trials in which the subject was distracted and moved too late (no movement before 800 ms) were eliminated from analysis (5.2% of trials).

### Statistical analysis

We hypothesized that exposure to startle would increase inappropriate flexor activity (faster and larger EMG flexor activities) which would negatively impact reaching movement (smaller distance, displacement and larger final error and slower movement onset). We used a Generalized Linear Mixed Effects model in R 2017 version 3.4.2 (Bates 2005) for all comparisons. Dependent variables included all outcome variables listed above (e.g. EMG onset, movement distance, etc.). The fixed effects were timepoint (beginning, end), target (Target 1, Target 2, Target 3), and muscle (BR, BIC, TRI, PEC, AD, PD). Subject was treated as a random factor and *P* < 0.05 was considered as statistical significance.

## Results

Voluntary trials showed increased distance and displacement, reduced movement onset, decreased final error and no changes in deviation from linearity at the end of the session. Additionally, muscle activity amplitude during voluntary trials did not change for most of the muscles during the session. Startle-evoked trials percentage, defined as the presence of SCM activity prior to 120 ms after the GO cue (SCM + %), was present during an average of 60.3 ± 8.8% of the SAS trials.

**Fig. 2:**
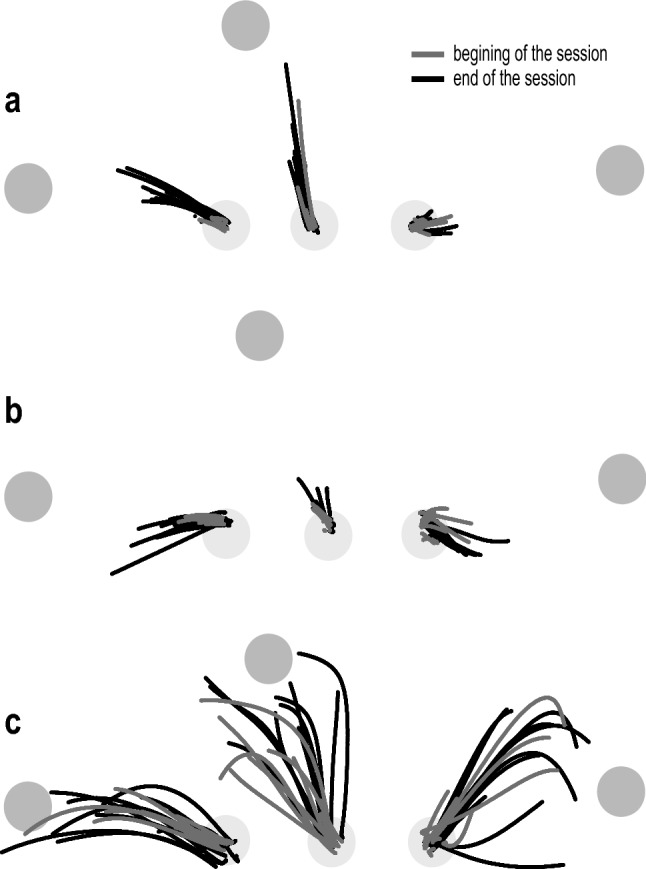
Beginning and end of the session reach trajectories for three representative subjects. The beginning trials in gray and the end trials in black are shown to each target for individuals with **a** UEFM = 11, MAS for: elbow flexion = 2, elbow extension = 2, shoulder flexion = 1, shoulder extension = 0, **b** UEFM = 19, MAS for: elbow flexion = 3, elbow extension = 3, shoulder flexion = 3, shoulder extension = 3, and **c** UEFM = 31, MAS for: elbow flexion = 2, elbow extension = 3, shoulder flexion = 0, shoulder extension = 0. *MAS* Modified Ashworth Scale, *UEFM* Upper Extremity Fugl–Meyer

At the end of the main session, subjects generated larger reaching movements towards the appropriate target (Fig. 2). Three representative subjects (Fig. 2) with varying levels of impairment and spasticity showed larger reaching distances at the end (black) compared to the beginning (gray) of the session. On average, at the end of the session, subjects generated voluntary reaches with 16.1% higher accuracy, 18.2% farther distance, 15.9% larger displacement, and 8.6% faster onset (faster onsets only present for Targets 1 and 2).

**Fig. 3:**
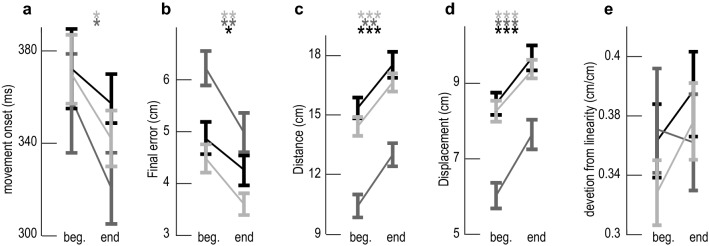
Group results of movement metrics. Movement onset (**a**), final error (**b**), distance (**c**), displacement (**d**) and deviation from linearity (**e**) for the beginning (left) and the end (right) of the session to Target 1 (light gray), Target 2 (dark gray) and Target 3 (black). *P < 0.05, **P < 0.01, ***P < 0.001 and the error bars are standard errors

Group results showed that final error was affected by timepoint (*F*_1,549_ = 21.25, *P* < 0.0001) and target (*F*_2,549_ = 19.13, *P* < 0.0001) leading to an average decrease of 0.83 ± 0.31 cm (Target 1: 0.86 ± 0.28 cm, *P* = 0.0019, Target 2: 0.99 ± 0.24 cm, *P* = 0.0017, Target 3: 0.64 ± 0.31 cm, *P* = 0.013) during the session (Fig. 3b). Distance was affected by timepoint (*F*_1,549_ = 32.20, *P* < 0.0001) and by target (*F*_2,549_ = 47.66, *P* < 0.0001) leading to an average increase of 1.93 ± 0.63 cm (Target 1: 2.38 ± 0.50 cm, *P* = 0.0001, Target 2: 1.24 ± 0.53 cm, *P* = 0.019, Target 3: 2.18 ± 0.63 cm, *P* = 0.0006) by the end of the session (Fig. 3c). Displacement was similarly affected by timepoint (*F*_1,549_ = 41.11, *P* < 0.0001) and by target (*F*_2,549_ = 51.03, *P* < 0.0001) leading to an average increase of 1.19 ± 0.30 cm (Target 1: 1.22 ± 0.30 cm, *P* = 0.0001, Target 2: 1.11 ± 0.27 cm, *P* = 0.019, Target 3: 1.23 ± 0.28 cm, *P* = 0.0001) by the end of the session (Fig. 3d). Movement onset was affected by timepoint (*F*_1,549_ = 4.88, *P* = 0.028) but not target (*F*_2,549_ = 1.90, *P* = 0.15) leading to decrease in onset for Target 1 (31 ± 15 ms, *P* = 0.014) and Target 2 (40 ± 21 ms, *P* = 0.039) but not Target 3 (23 ± 19 ms, *P* = 0.35) by the end of the session (Fig. 3a). Finally, the deviation from linearity did not change significantly for any of the targets (*P* > 0.1 for all the targets) during the session (Fig. 3e).

**Fig. 4:**
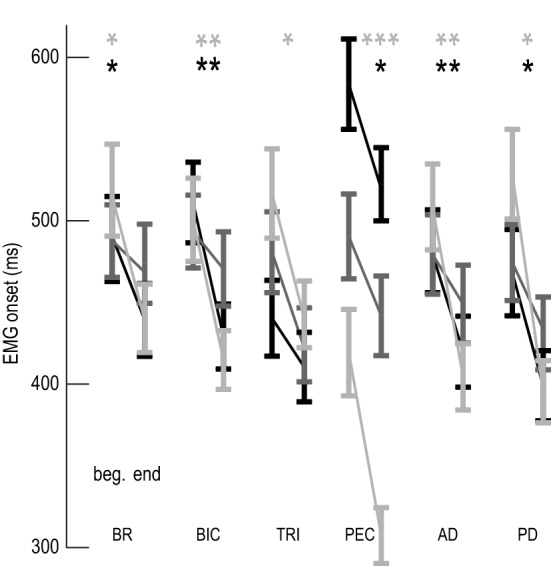
Group results of muscle activity onset. EMG onset for the beginning (left) and the end (right) of the session to Target 1 (light gray), Target 2 (dark gray) and Target 3 (black) for each muscle. *BR* brachioradialis, *BIC* biceps, *TRI* triceps lateral head, *PEC* pectoralis, *AD* anterior deltoid, *PD* posterior deltoid. *P < 0.05, **P < 0.01, ***P < 0.001 and the error bars are standard errors

Group results showed that muscle activity onset was affected by timepoint (*F*_1,3573_ = 86.88, *P* < 0.0001), target (*F*_2,3573_ = 13.61, *P* < 0.0001) and muscle (*F*_5,3573_ = 5.67, *P* < 0.0001) (Fig. 4). Muscle onset was faster for all muscles at Target 1 (avg Δ = 92 ± 31 ms, all: *P* < 0.013), none of the muscles at Target 2 (all: *P* > 0.09), and all muscles except TRI for Target 3 (avg Δ = 69 ± 27 ms, all: *P* < 0.02; TRI: *P* = 0.27) (Fig. 4).

**Fig. 5:**
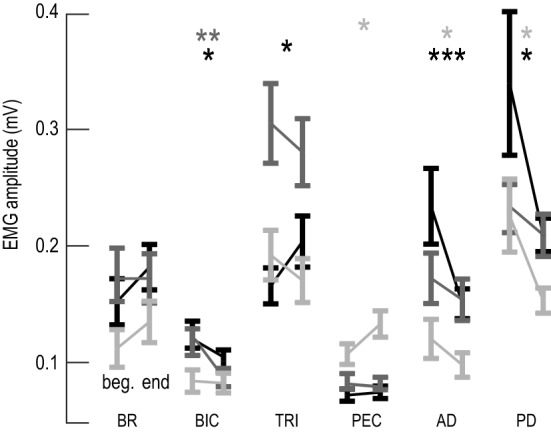
Group results of muscle activity amplitude. EMG amplitude for the beginning (left) and the end (right) of the session to Target 1 (light gray), Target 2 (dark gray) and Target 3 (black) for each muscle. *BR* brachioradialis, *BIC* biceps, *TRI* triceps lateral head, *PEC* pectoralis, *AD* anterior deltoid, *PD* posterior deltoid *P < 0.05, **P < 0.01, ***P < 0.001 and the error bars are standard errors

Muscle activity amplitude was affected by timepoint (*F*_1,3573_ = 3.75, *P* = 0.05), target (*F*_2,3573_ = 12.34, *P* < 0.0001) and muscle (*F*_5,3573_ = 200.2, *P* < 0.0001). For Target 1, the shoulder horizontal adduction target, activity was increased in PEC (Δ = 0.026 ± 0.01 mV, *P* = 0.048) and decreased in AD (Δ = 0.02 ± 0.02 mV, *P* = 0.037) and PD (Δ = 0.073 ± 0.03 mV, *P* = 0.045). For Target 2, the combination elbow extension and shoulder flexion target, BIC activity was decreased (Δ = 0.030 ± 0.01 mV, *P* = 0.007). For Target 3, elbow extension task, TRI activity was increased (Δ = 0.038 ± 0.02 mV, *P* = 0.033), while BIC (Δ = 0.023 ± 0.01 mV, *P* = 0.049), AD (Δ = 0.083 ± 0.03 mV, *P* = 0.0004) and PD (Δ = 0.13 ± 0.06 mV, *P* = 0.042) were decreased (Fig. 5).

## Discussion

It has been demonstrated that startle can improve movement parameters in iwS (Honeycutt et al. 2015; Rahimi and Honeycutt 2020; Honeycutt and Perreault 2012, 2013; Coppens et al. 2018; Ossanna et al. 2019); however, startle also induces task-inappropriate flexor activity (Rahimi and Honeycutt 2020; Honeycutt and Perreault 2012, 2014) raising concerns that exposure to startle might increase inappropriate activity during voluntarily initiated movements. For example, the severe group from this dataset had early flexor activation preceding triceps (TRI), while initiating an extension task, limiting reaching distances during startle-evoked trials (Rahimi and Honeycutt 2020). Thus, the objective of this study was to evaluate the impact of exposure to startle on voluntary movements (non-startle-evoked) during point-to-point reaching in individuals with moderate and severe stroke. We found that while abnormal flexor activity was present in startle-evoked trials during point-to-point reaching in all three directions (Rahimi and Honeycutt 2020; Honeycutt and Perreault 2014), voluntary movements did not see an increase in abnormal flexor activity. In opposition to our hypothesis, agonist muscle activity was increased and task-inappropriate, antagonist flexor activity decreased. Moreover, exposure to startle led to a small increase in subjects’ ability to reach farther, start reaching faster, and more accurately. While this represents a short exposure period (~ 1 h), it suggests that at least short-term exposure to startle does not lead to facilitation of inappropriate flexor synergies, but rather may reduce them. Future studies are required to determine if this effect will facilitate larger reaching movements in comparison to control groups.

We predicted larger EMG amplitude in flexor muscles during the voluntary trials as the impact of the startle trials. However, our results showed that this prediction was not true. For Target 1, a shoulder horizontal adduction task, we showed EMG amplitude increases in the PEC muscle, paired with decreases in PD extensor activity in voluntary trials. This is beneficial, as more shoulder horizontal adduction can occur with decreased antagonist extension activity from PD. For Target 2, an elbow extension and shoulder flexion task, we showed a similar decrease in BIC activity during voluntary trials (Fig. 5). The decrease in BIC is further evidence that abnormal flexor activity did not affect Targets 2 and 3, with biceps being a common culprit for post-stroke spasticity (Li et al. 2014; Choudhury et al. 2019). Lastly, for Target 3, an elbow extension task, appropriate increases in triceps activity coincide with decreases in BIC, AD, and PD activity during voluntary trials. Moreover, there was an unexpected increase for TRI (an extensor) for Target 3, allowing for improved reaching distances (Fig. 5). In short, flexor EMG amplitudes seem to decrease in the majority of cases for voluntary movements after a SAS session, while extensors (TRI) increase.

This study offers the first evidence that exposure to startle in severe-to-moderate iwS is safe and does not lead to increases in task-inappropriate flexor activity during voluntary movement. Further study is required before we can determine if this can be used for clinical relevance. More robust evaluation of muscle activity via an analytical synergy analysis method (e.g. nonnegative matrix factorization) (Avella and Bizzi 2005; Tresch et al. 1999) and under conditions where abnormal flexor activity is highest (arm supported against gravity) (Ellis et al. 2016, 2017; Sukal et al. 2006) is warranted.

### Startle for use in rehabilitation

While further controlled studies are needed to confirm that exposure to startle can lead to increases in reaching distance, the results from this study that exposure to startle does not increase abnormal flexor synergies in individuals with severe/moderate stroke may open the possibility of startle in rehabilitation. Contrary to our hypothesis, the movement distance, onset, displacement, and final accuracy increased for most or all targets as a result of this task. Therefore, exposure to startle did not lead to abnormal flexor activity in voluntary movement, but instead led to decreases in flexor (BIC, AD, PD) EMG amplitudes, and increases in extensors (TRI). This led to an average change of 1.93 ± 0.63 cm in reaching distance, which constitutes an 18% change—larger than what similar studies have reported after longer and more frequent rehabilitation sessions for chronic, severe stroke (McPherson et al. 2018a; Dean and Shepherd 1997; Raghavan et al. 2017; Aşkın et al. 2018; Barker et al. 2008), suggesting that startle exposure may decrease flexion. Our results indicate that startle exposure does not increase abnormal flexor synergies and future studies should determine if we can use startle exposure appropriately in clinical settings.

There are different ways to quantify minimum detectable change (MDC). Several tests for reaching distance [e.g. Functional Reach Test (Katz-Leurer et al. 2009)] do not account for compensatory trunk movements during reaching. Mandon et al. (2016) found no significant differences in reaching distance during trunk constraint vs. no trunk constraint for the Action Research Arm Test (ARAT), which defines MDC as 10% of the maximum target distance (in this study, MDC would be 1.1 cm). Wagner et al. (2008) uses two methods to quantify reach extent while accounting for trunk movements. First phase, which defined end of movement as when tangential wrist velocity drops to a minimum before secondary corrections yielded an MDC of 26.6%; and percentage of peak hand velocity (% PHV), which instead defines end of movement as when the wrist velocity drops below 5% of the peak yielded an MDC of 12%. Wagner et al. report that the % PHV method produced greater reproducibility for measuring reach extent. When that threshold (12%) is used, we show changes for displacement and distance that exceed that threshold across the board (Target 1: 15.4 and 14.7%; Target 2 24.9 and 18.4%; Target 3: 18.2 and 15.9%; Average across targets: 18.2 and 15.9%). These minimum detectable changes are also supported by the ARAT’s threshold of 1.1 cm, provocatively suggesting that startle exposure might induce functionally significant changes. Previous studies evaluating individuals with chronic stroke show small improvements after numerous sessions, highlighting the challenge in making functional changes in individuals with severe/moderate stroke (McPherson et al. 2018a; Dean and Shepherd 1997; Raghavan et al. 2017; Aşkın et al. 2018; Barker et al. 2008). Recent studies with a minimum of 10 sessions and therapies ranging from seated training to virtual reality report at most an 11% increase in reaching distances and a 4% increase of range of motion—even when these novel therapies were paired with conventional physical therapy (McPherson et al. 2018a; Dean and Shepherd 1997; Raghavan et al. 2017; Aşkın et al. 2018; Barker et al. 2008). Future controlled studies should evaluate if startle exposure during traditional therapy might enhance reaching in individuals with severe/moderate stroke.

If startle is shown to generate functionally significant changes, understanding the mechanisms will become more important. It is possible that exposure to startle may result in reliance on the brainstem or, given the short-term facilitation, activation of these muscles releases spasticity. The early flexor activation we report (Rahimi and Honeycutt 2020) during startle-evoked trials may result from an interfering hypermetric classic startle response (Rahimi and Honeycutt 2020; Honeycutt and Perreault 2012; Choudhury et al. 2019). Not only do iwS have increased ipsilateral projections of the reticulospinal tract (Karbasforoushan et al. 2019; Herbert et al. 2015), but the cortex, which likely mediates the amplitude of a classic startle reflex (Davis and Gendelman 1977; Honeycutt and Perreault 2014), is damaged post-stroke and can no longer fully suppress a classic startle reflex. This likely causes task-inappropriate flexion when iwS’ movements are paired with a startle, while in healthy individuals, startle only elicits the planned movement (Ossanna et al. 2019; Kirkpatrick et al. 2018; Carlsen et al. 2011). During voluntary trials that are not preceded by a startle, the subject can initiate their movement uninterrupted by classic startle, while also becoming more practiced in the task. An alternative mechanism may be that startle over-activates flexor muscles, leading to decreases in neglect-related spasticity. When startle is used to elicit movement in iwS, flexor activity surpasses the maximum voluntary contraction (MVC) in individuals with severe stroke by 2–3 times (Rahimi and Honeycutt 2020). This over-activation of paralyzed muscles may mimic the effects of functional electrical stimulation (FES), which decreases spasticity and increases range motion (Rabadi and Aston 2017). While the mechanisms driving this success are not fully understood, electrical activation of muscle may free paralyzed cross-bridge attachments and allow infiltration of ions associated with muscle contraction, “releasing” the spastic muscles (Rabadi and Aston 2017). It is reasonable to expect that startle generates a similar outcome, given the over-activation achieved. In conclusion, future studies should evaluate both neuroplastic modulation of the brainstem as well as spasticity release as potential mechanisms driving startle-related changes in movement—both startle-evoked and voluntary.

### Limitations and future directions

Though this study demonstrates that short-term exposure to startle does not lead to facilitation of inappropriate flexor synergies, whether or not it *decreases* abnormal patterns of activity (leading to enhanced extension) is still unclear. The reaching distance improvements seen here could simply be the result of practice over the session. Future controlled studies should include a control group to evaluate this effect. Additionally, the voluntary trials analyzed at the beginning and end of the session were selected from blocks that included 5 SAS trials. Future studies should ideally include have voluntary trials that have not been contaminated by SAS trials, but instead be performed as blocks without any startling stimuli.

Above we report that all targets showed a functionally relevant change in reaching distances and displacements as defined by one of the analysis methods used (% PHV) by Wagner et al. (2008). However, none of the targets reach the threshold (26.6%) when minimal detectable change is defined by the authors’ other analysis method (first phase). Although the authors report that the % PHV method produced greater reproducibility for measuring reach extent, we recognize that the functional significance we claim here is not confirmed by both analysis methods. This could result from small sample size and a mere 11 cm target distance; however, we also see improvements in accuracy, with final error showing an average decrease of 0.83 ± 0.31 cm (all targets *P* < 0.013). Additionally, the minimal detectable change was defined using a different setup with a larger workspace (Wagner et al. 2008), which allowed for more freedom of movement. Here, participants were limited by a table to constrain movements to two dimensions. Future studies evaluating this question should address the size of the workspace so that movements are not as limited, but the fact that we see such large changes despite these limits is promising.

## Conclusion

In conclusion, this study offers the first evidence that exposure to startle in iwS does not negatively impact voluntary movement, but that exposure may improve volitionally activated reaching movements by decreasing abnormal flexion activity. This result indicates that at least short-term exposure to startle is safe and opens up the possibility of startle being used for rehabilitation.
